# Depression and adherence to antiretroviral treatment in HIV-positive men in São Paulo, the largest city in South America: Social and psychological implications

**DOI:** 10.6061/clinics/2017(12)05

**Published:** 2017-12

**Authors:** Ricardo Pereira de Moraes, Jorge Casseb

**Affiliations:** I Ambulatorio de Imunodeficiencias Secundarias, Departamento de Dermatologia, Hospital das Clinicas HCFMUSP, Faculdade de Medicina da Universidade de Sao Paulo, Sao Paulo, SP, BR; II Instituto de Medicina Tropical de Sao Paulo, Sao Paulo, SP, BR

**Keywords:** Adherence, Depression, Epidemiology, Ethnicity/Race, Gender

## Abstract

**OBJECTIVES:**

The aim of the present study was to investigate the prevalence of depression and adherence to antiretroviral treatment in two groups of individuals: men who have sex with men (MSM) and men who have sex with women (MSW).

**METHODS:**

Two hundred and sixteen participants (MSM=116; MSW=100) who visited the Clinics Hospital of the School of the Medicine of the University of São Paulo completed two independent surveys (the BECK Depression Inventory and an adherence self-declared questionnaire) to evaluate their depression status and adherence to antiretroviral treatment, respectively.

**RESULTS:**

The study highlighted a positive relationship between depression and low adherence to Highly Active Antiretroviral Therapy in these patients regardless of age and sexual orientation. In addition, MSM subjects were two times more prone than MSW subjects to develop depression symptoms. White or mixed race men showed 7.6 times greater adherence to treatment than black men. The probability of complete adherence to treatment was 3.8 times higher in non-depressed subjects than in depressed subjects regardless of their ethnicity.

The chance of developing depression was 4.17 times higher for an individual with non-adherent behavior than for an adherent individual.

**CONCLUSIONS:**

Individuals with low adherence rates have proportionally higher depression rates. Depressed men tend to show less adherence to treatment. Black but not mixed race or white men show less adherence to Highly Active Antiretroviral Therapy and have a greater chance of developing depression, which directly interferes with adherence. The chances of developing depression are four times greater for a patient with non-adherent behavior than for a patient with adherent behavior.

## INTRODUCTION

Globally, HIV/AIDS is a serious public health concern. Brazil faces severe social implications due to an estimated HIV-infected population of 700,000. Historically, two important facts underscore the calamity of the AIDS epidemic—in 1995, AIDS was the cause of the highest number of deaths worldwide, and the largest number of new HIV cases was diagnosed in 1997. Due to a great deal of effort by the international community and the availability of advanced medical care for infected patients, the infection rate has largely stabilized. At present, HIV is considered a chronic condition that requires a continuous and prolonged use of drugs 1.

Adherence to treatment and maintaining a relationship with health services are key indicators of the success of the medical management of chronic diseases 2. The degree of adherence to treatment depends on the extent to which the patient understands and agrees with the provided guidelines. In cases with low adherence, the patient may develop opportunistic infections and experience daily discomfort or recurrent episodes of minor viral infections, leading to poor consequences in the individual’s life. An emerging major public health problem is the possibility of transmission of a multidrug-resistant virus 2.

Several authors have shown that poor adherence to drug therapy has significantly reduced the well-known clinical benefits for HIV-infected individuals, leading to a complete failure of treatment and the patient’s death. Following 95% of the proposed treatment scheme is the desired level of adherence 3,4,5.

Many studies have noted the need to monitor patients’ adherence statuses. The initial phase of treatment is considered the most critical period for successful adherence. Therefore, approaches including individualized care and constant monitoring of the patient by a multidisciplinary team are recommended 6.

Currently, 91% of adherence studies have been performed in the United States and Europe, and few studies have reported data from Brazil. Even the sparse Brazilian reports suffer from limitations, because they have not been conducted in accordance with international standards 5,7. Therefore, monitoring adherence to antiretroviral treatment at referral centers is an important method to identify those at high risk for treatment failure and provide an early intervention 6,2.

Drug efficacy is also determined by various psychological factors. Depression, which is a common condition that affects more than 350 million people worldwide according to the World Health Organization, is directly associated with non-adherence to HIV treatment 8,9 and is expected to be the third leading cause of disability worldwide by 2020 10. Depression is a common mental health problem that is associated with a decreased quality of life, productivity loss, and family stress (Reviewed by 11). Depressive symptoms are often managed by primary care physicians unless the severity of the condition requires specialist psychiatric care (Reviewed by 11). Some common features of depression include the presence of an irritable mood accompanied by somatic and cognitive changes that significantly affect the individual’s capacity to function 12. Depression is characterized by general feelings of sadness, anhedonia, avolition, worthlessness, and hopelessness. Cognitive and neurovegetative symptoms, such as difficulty concentrating, memory alterations, anorexia, and sleep disturbances, have also been reported 12.

The high level of correlation between depression/suicide and depression/non-adherence to treatment is disturbing for care providers. Studies have suggested that depression is the most common psychological disorder affecting HIV-infected individuals 13. Moreover, depression and anxiety affect a patient’s behaviors and consequently their adherence to treatment due to apathy, hopelessness, self-neglect, and forgetfulness, which aggravates the scenario 14,15,16.

Failure to adopt the treatment in a proper manner has been identified in HIV-positive individuals reporting at least one episode of depression, which increases their chances of developing further health problems 17 and risky behaviors 13,18. Globally, people living with HIV/AIDS are affected by depression two times as often as the general population 9,19,20.

In addition, the frequency of depressive episodes has a direct correlation with the chances of non-adherence to antiretroviral therapy 21. Changes in an individual’s behaviors due to depression can also disrupt interpersonal relationships and social networks, which negatively influences the immune status and productive lifestyle and considerably increase welfare-related problems 8,22.

Due to the scarce data available on the relationship between depression and adherence to antiretroviral treatment in Brazil, the present study aimed to investigate the prevalence of depression among male HIV/AIDS patients classified as men who have sex with men (MSM) and men who have sex with women (MSW) in São Paulo city, which is the most developed region of Brazil.

## MATERIALS AND METHODS

### Participants

A total of 216 men with HIV attending the Clinics Hospital of the School of Medicine of the University of São Paulo participated in the study. All participants were referred for follow-up treatment by the local blood bank (Fundação Pró-Sangue Hemocentro de São Paulo) after a positive serology test for HIV. The data collection period was between March 2014 and December 2015.

### Procedure

A cohort of 304 HIV-positive men in active follow-up at the Clinics Hospital of the Medical School of the University of São Paulo was invited to participate in this study. Two hundred and sixteen participants (71.05%) agreed to participate after meeting the following inclusion criteria: HIV status; minimum age of 18 years; and use of antiretroviral drugs. The participants were divided into two groups (MSM and MSW).

### Ethics Committee

The study was approved by the Ethics Committee of the Clinics Hospital of the School of Medicine of the University of São Paulo (CAPPesq, number 473.230). The participants consented to the use of survey data in the present analysis.

### Questionnaires

The Beck Depression Inventory was applied to identify possible depression symptoms among all subjects. The Beck inventory is a self-administered questionnaire that consists of 21 groups of statements. The inventory, which was previously validated for use among the Brazilian public, enables the construction of a depression score for each participant based on a self-report 23,17,18. To evaluate adherence, one inventory was applied with functions similar to the inventories adopted for the START (“The Strategic Timing of Antiretroviral Treatment Study”) and SMART 19,20 studies 24.

### Statistical analysis

Descriptive statistics with means and standard deviations (SDs) were reported for continuous variables, and frequencies were reported for categorical variables. A two-way contingency analysis (*χ^2^*) was performed to assess whether a categorical variable significantly differed between the two groups (MSM and MSW), and Student’s t-test was performed to compare continuous variables between the groups. The statistical analysis was performed using logistic regression for adherence and analysis of categorical data for depression. The following variables were collected from each participant and tested for both models: age group (40 years or younger and 41 years or older); schooling (in years); ethnicity (white, mixed, or black); viral load; CD4^+^ T lymphocyte count; marital status (married, single, separated, or divorced); sexual orientation (MSM or MSW); and mode of transmission of disease (sexual contact with a person of the same sex or with a person of the opposite sex, blood products, or others). A model of the linear log class was used within the categorized data analysis to estimate the probability of depression. Initially, one linear log model was built to check which variables were associated with the depression level. Subsequently, the most parsimonious model was achieved using adherence and age as variables. The model’s goodness of fit (and that of the intermediate models resulting from models with feature removal) was measured using statistical ward continuity.

Regarding the adherence model, logistic regression was used to analyze the association between adherence to treatment and other variables. All features were initially included in the model, and the final and most parsimonious model was achieved using stepwise selection with depression and ethnicity as its variables. The quality of adjustment of the model was checked using a residual envelope graph.

After quality adjustment for both models using a statistical ward (categorical data) and envelope graph (logistic regression), the final analysis was performed using the R Studio software.

The results of both models were expressed and interpreted through odds ratios (ORs) and 95% confidence intervals (CIs) for all features whether or not they were statistically significant. The level of significance for all statistical tests was set at 0.05.

### Electronic data management

The database was developed using the Research Electronic Data Capture (REDCap) tool hosted in our institution’s (School of Medicine, University of São Paulo) web server. This tool allowed validation, auditing, and export of the study data^25^.

## RESULTS

### Demographic and clinical laboratory data

Among the 216 HIV-infected men, 116 (53.7%) were MSM and 100 (46.3%) were MSW (Table 1). The ethnicities of the subjects were as follows: 70.4% white; 17.6% mixed; 8.8% black; and 3.2% unknown (Table 1). The majority of the participants in both groups were over 40 years of age. No significant differences in ethnicity and age were observed between the MSM and MSW. Similarly, schooling was identical for both groups.

However, significant differences were found in the marital status (*p*<0.001), with 86.2% of the MSM and 38.0% of MSW single and 5.1% of the MSM and 50.0% of the MSW married (Table 1).

The transmission of infection occurred chiefly through sexual intercourse with persons of the same sex (43.6%, with rates of 73.3% and 9% for MSM and MSW, respectively), whereas 19.0% of the infections were acquired via sexual intercourse with persons of the opposite sex (3.4% and 37% for MSM and MSW, respectively) (Table 1). A higher percentage of individuals in the MSW group than in the MSM group did not know how they acquired the infection (Table 1).

Clinical data indicate that 52.8% of all participants had CD4^+^ lymphocyte counts >500 mm^3^, and 18.1% had counts <200 mm^3^ (Table 1). The viral load was undetectable or <10,000 copies in 95.4% of the participants (Table 1).

### Depression and adherence

The Beck questionnaire revealed that 32.5% of the participants in both groups had some level of depression (Table 2). Importantly, 8.4% of the participants presented severe symptoms of depression (Table 2). Almost all participants (90.8%) declared adherence to the antiretroviral therapy (Table 2).

### Depression as a variable response

After dividing the variable response (depression) into four categories (minimum or none, mild, moderate, and severe), the probability of each category was estimated based on the explanatory variables (see Table 3).

Table 3 shows the results of the final log-linear class model from the categorized data analysis. The category of depression used as a reference was minimum or none.

Variables such as adherence (*p*=0.046), age (*p*=0.042), and transmission (*p*=0.076) showed a greater correlation with depression in the multivariate analysis. Since the sample size might not be representative of all categories of depression, considering *p*=0.07 statistically significant was acceptable.

The results showed that men younger than 40 years had a 2.9-fold higher chance of developing moderate depression than older men. Men who acquired the disease due to sexual contact with persons of the same sex had a 2.0-fold greater chance of developing severe depression than men who acquired the disease through sexual contact with the opposite sex. Those reporting poor adherence to treatment had a 3.8-fold higher chance of suffering from moderate depression than those reporting complete adherence. The results are summarized in Tables 3 and 4.

**Figure 1:**
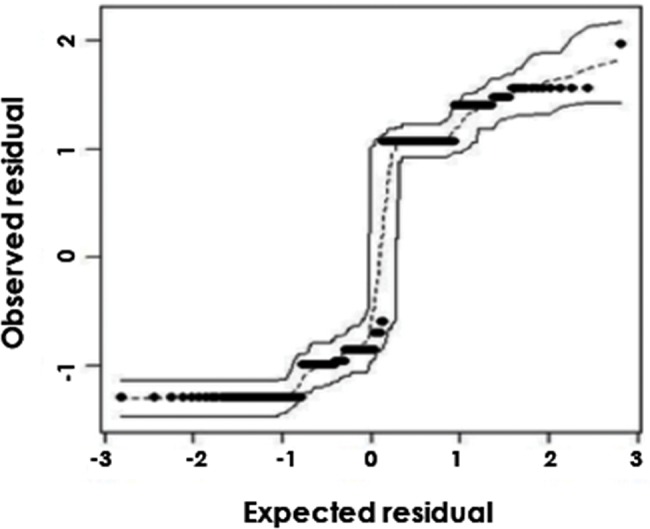
The diagnosis of the logistic regression model. Since all residues are within the confidence band of the expected distribution, the model is well adjusted.

The goodness of fit of the model was validated using statistical ward continuity (*p*=0.73). This test verified our hypotheses and proved that the model was adequate, with a high *p-*value indicating that the residues followed a normal distribution with a mean of 0 and variance of 1 (Figure 1).

### Adherence as a variable response

The adherence and non-adherence parameters were analyzed by logistic regression. The probabilities were estimated for each category by considering other variables (age, viral load, CD4^+^ T lymphocyte count, marital status, education, HIV transmission, and sexual orientation). The parameters were shortened and validated through successive tests of the likelihood ratio (with *p*=0.05). The results of the model showed that ethnicity (*p*=0.0008) and depression (*p*=0.0134) were significantly related to adherence, whereas the relationships of the other variables were not significant [age (*p*=0.33), viral load (*p*=0.26), CD4^+^ T lymphocyte count (*p*=0.5), marital status (*p*=0.47), education (*p*=0.53), transmission (*p*=0.28), and sexual orientation (*p*=0.37)] (Table 5).

The distribution of adherence based on ethnicity and depression is shown in Table 6. Accordingly, white or mixed race men with depression reported 90.6% adherence to antiretroviral therapy, whereas black men with depression reported 33.3% adherence to treatment. Our statistical analysis showed that white or mixed race men were 7.7 times more likely to adhere to treatment than black men. The probability of a non-depressed subject adhering completely to the treatment was 3.8 times higher than the probability of depressed subjects adhering to treatment regardless of ethnicity.

## DISCUSSION

The goal of our study was to evaluate the prevalence of depression in men with HIV/AIDS who had sex with men (MSM) or women (MSW) and its relationship with treatment adherence. Our results showed that adherence did not depend on age or sexual orientation; the overall adherence rate in this study was 90.74%. Ninety-five percent of non-depressed men adhered to treatment, whereas 86% of depressed individuals showed adherence. Black men showed less adherence (68%) than white (93%) and mixed race men (97%). Regardless of sexual orientation, the non-adherents were 2.84 times more likely to develop depression. Thus, depression has a direct negative action on adherence.

Depression was the most commonly reported psychological disorder among our participants. In addition, being in a constant state of depression might influence immune responses 9,21,26. Notably, this disorder is largely underdiagnosed in this population 27. Depression in an HIV-infected individual has been reported to largely hinder the patient’s life. In a study with 55 individuals, Porche and Willis (2006) found that 64% of the participants were diagnosed with depression, although only 42% reported symptoms 28. The most common symptoms of depression reported in our survey were in agreement with studies from different countries 29,30.

Investigations worldwide have revealed that the prevalence of depression is two to three times higher in HIV-positive individuals than in the general population 31,33.

The depression rates in our sample were comparable to those reported in other studies. However, no significant differences were found between the groups regarding sexual orientation, although the MSM group showed a higher tendency for depression since they predominantly contracted the disease by sexual contact with people of the same sex. One main novel element in this study was that although previous studies compared the state of depression between groups of patients with HIV and those without 34, this study performed a comparison between two subgroups of HIV-infected individuals. Our results suggested that sexual orientation had no significant influence on depression, whereas age and adherence had detrimental effects on moderate depression symptoms. Married men showed a lower rate of depression than the other men. In line with this finding, the group with the highest prevalence of depression was divorced individuals. Importantly, single men who were infected through intercourse with same-sex partners showed two times more severe depression symptoms than men who were infected through intercourse with partners of the opposite sex.

Previous studies have shown that HIV-infected women have higher levels of depression than HIV-infected men 15,22,35,36. A survey in Brazil reported by Mello et al. (2006) indicated that the prevalence of depression in HIV-infected women ranged from 25% to 45%. Interestingly, the percentage of depression observed in the MSM group in our study was comparable to that reported for HIV-positive women in other studies 35,33,29.

Depression is widely thought to reduce the degree of adherence to antiretroviral therapy 37,38. In contrast, our results indicated that even depressed patients showed adequate adherence to antiretroviral treatment.

Studies have confirmed that good adherence rates are directly related to medical assertiveness, which encourages the patient to establish a good relationship with health professionals 24,39,40.

Different cohorts of HIV-infected patients reported an average of 65.6% adherence to Highly Active Antiretroviral Therapy 41,42. Similarly, in the present study, we observed a high rate of adherence to antiretroviral treatment. We noted that depression and adherence did not show any correlation with sexual orientation. Another study reported that no significant relationship existed between depression and non-adherence to antiretroviral treatment 37. The high rates of adherence among both groups can be attributed to the excellent services offered by health professionals in our care setting. Most patients in our cohort were under follow-up care for a long time, which suggested that a consistent relationship was a decisive factor for the success of antiretroviral treatment. Many other studies have reached a similar conclusion 43,31.

The likelihood of adherence to antiretroviral treatment is 83% higher if symptoms of depression are identified and care is provided. The risk of non-adherence increases by 35% among those who do not receive any treatment for depression 30.

We observed a significant relationship between depression and adherence. Most of the non-adherent patients presented only milder forms of depression, because the multidisciplinary treatment approach in our institution quickly and appropriately addressed the psychological symptoms of depression in patients.

Regarding the ethnicities of the patients, we found that black men were less adherent to treatment than white and mixed race men. The probability of non-depressed white or mixed race men adhering to treatment was 96.7%; however, the probability of adherence decreased to 88.5% in depressed individuals. Conversely, the probability of adherence in non-depressed black men was 79.2%, whereas the probability decreased to 49.9% in depressed black men. These results corroborate those of other studies showing black men have lower adherence 44,45 and less of a propensity toward adherence with antiretroviral therapy 46.

Studies on African-American men have indicated that stigma, poor reliability on medical assistance, poor social conditions, and psychosocial issues negatively impact the care inherent for those with HIV-positive conditions 47,4.

Unfortunately, our study data did not consider the economic statuses of the patients for analysis. Consideration of the economic statuses would have allowed a better interpretation of our findings on the population of African Brazilian men living with HIV/AIDS.

The relationship between age, adherence, and moderate depression was significant in our patient groups.

To the best of our knowledge, our study is the first to report the prevalence of depression and adherence to antiretroviral treatment in HIV-positive men in São Paulo, which is the largest city in South America. Our report showed evidence that black men living with HIV/AIDS who had same-sex partners needed more attention from a multidisciplinary team regarding their depression statuses and reduced adherence to antiretroviral treatment.

## AUTHOR CONTRIBUTIONS

de Moraes RP was responsible for the research procedures and manuscript writing. Casseb J was the research supervisor.

## Figures and Tables

**Table 1:** Demographic data from 216 patients infected by HIV.

Variables	MSM	MSW	Total	*p-value	CI 95% *(msm – msw)*
**Mean age**	44.6±11.98	48±10.39	46.2±11.37	**0.026**	[-6.38; -0.42]
**Age in years**					
≤40	39 (33.6%)	19 (19%)	58 (26.9%)	**0.024**	[2.2%; 27.1%]
≥41	77 (66.4%)	81 (81%)	158 (73.1%)	**0.024**	[-27.1%; -2.2%]
**Ethinicity**					
White	85 (73.3%)	67 (67%)	152 (70.4%)	0.39	[-7.0%; 19.4%]
Mixed	20 (17.2%)	18 (18%)	38 (17.6%)	1	[-11.7%; 10.2%]
Black	11 (9.5%)	8 (8%)	19 (8.8%)	0.87	[-6.9%; 9.9%]
W/information	0 (0%)	7 (7%)	7 (3.2%)	**0.004**	[-12.9%; -1.1%]
**Marital Status**					
Single	100 (86.2%)	38 (38%)	138 (63.9%)	**<0.001**	[35.9%; 60.5%]
Married	6 (5.1%)	50 (50%)	56 (25.9%)	**<0.001**	[-56.4%; -33.3%]
Separated	1 (0.9%)	5 (5%)	6 (2.8%)	0.09	[-9.7%; 1.4%]
Divorced	8 (6.9%)	3 (3%)	11 (5.1%)	0.32	[-2.7%; 10.5%]
W/information	1 (0.9%)	4 (4%)	5 (2.3%)	0.18	[-8.3%; 2.0%]
*Education in years					
until 05	8 (6.9%)	9 (9%)	17 (7.9%)	0.75	[-10,3%; 6,1%]
until 09	36 (31.1%)	40 (40%)	76 (35.2%)	0.22	[-22,7%; 4,7%]
more than 10	62 (53.4%)	41 (41%)	103 (47.7%)	0.09	[-1,7%; 26,6%]
W/information	10 (8.6%)	10 (10%)	20 (9.2%)	0.91	[-10,0%; 7,3%]
**Transmission**					
*S.C.W.S.S.P.	85 (73.3%)	9 (9%)	94 (43.6%)	**<0.001**	[53.5%; 75.0%]
*S.C.W.O.S.P.	4 (3.4%)	37 (37%)	41 (19%)	**<0.001**	[-44.5%; -22.6%]
Blood	0 (0%)	3 (3%)	3 (1.4%)	0.10	[-7.3%; 12.7%]
Others	1 (0.9%)	4 (4%)	5 (2.3%)	0.18	[-8.3%; 2.0%]
Unknown	18 (15.5%)	32 (32%)	50 (23.1%)	**0.007**	[-28.6%; -4.3%]
W/information	8 (6.9%)	15 (15%)	23 (10.6%)	0.09	[-17.4%; 1.2%]
**Lymphocytes T CD4^+^**					
<200	19 (16.4%)	20 (20%)	39 (18.1%)	0.60	[-14.9%; 7.6%]
200 - 350	7 (6%)	6 (6%)	13 (6%)	1	[-6.4%; 6.4%]
350 - 500	30 (25.9%)	20 (20%)	50 (23.1%)	0.39	[-6.3%; 18.0%]
>500	60 (51.7%)	54 (54%)	114 (52.8%)	0.84	[-16.6%; 12.0%]
**Viral load HIV**					
0 - 10.000	112 (96.6%)	94 (94%)	206 (95.4%)	0.52	[-4.1%; 9.2%]
10.000 - 100.000	3 (2.6%)	5 (5%)	8 (3.7%)	0.48	[-8.5%; 3.7%]
>1000.000	1 (0.8%)	1 (1%)	2 (0.9%)	1	[-2.9%; 2.6%]

* The *p*-values (Fisher Exact test) indicated here indicate whether HSM / MSM populations are different in age, ethnicity, marital status, education, transmission, lymphocytes T CD4+ and viral load. There is no relation to the response variables (adherence and depression).

*in education item in this table, divided by educational cycles practiced in Brazil.

*MSM = Men who have sex with men

*MSW= Men who have sex with women

*S.C.W.S.S P.= Sexual contact with same sex partner

*S.C.W.O.S.P. = Sexual contact with opposite sex partner.

**Table 2:** Absolute numbers and respective percent of depression by Beck depression inventory (BDI) and adherence data by START questionnaire.

B.D.I.	MSM	MSW	All	*p-value	CI 95% *(msm – msw)*
0 - 13	73/ (62.9%)	73/ (73%)	146/ (67.5%)	0.15	[-23.3%; 3.2%]
14 - 19	22/ (18.9%)	13/ (13%)	35/ (16.2%)	0.32	[-4.7%; 16.6%]
20 - 28	12/ (10.3%)	5/ (5%)	17/ (7.9%)	0.23	[-2.5%; 13.3%]
29 - 63	9/ (7.7%)	9/ (9%)	18/ (8.4%)	0.93	[-9.5%; 7.1%]
**Adherence**					
All	106/ (91.4%)	90/ (90%)	196/ (90.8%)	0.91	[-7.3%; 10.1%]
Majority	7/ (6.0%)	6/ (6.0%)	13/ (6.0%)	1	[-6.4%; 6.4%]
Half	2/ (1.7%)	1/ (1.0%)	3/ (1.4%)	1	[-3.1%; 4.5%]
Few	0	2/ (2.0%)	2/ (0.9%)	0.21	[-5.6%; 1.7%]
None	1/ (0.9%)	1/ (1.0%)	2/ (0.9%)	1	[-2.8%; 2.6%]

B.D.I.

0 - 13 - Minimum depression

14 - 19 - Mild depression

20 - 28 - Moderate depression

29 - 63 - Severe depression.

ADHERENCE

All - Ingestion of all prescribed doses

Majority - Ingestion of most prescribed doses

Half - half doses intake

Few - Ingestion of a few doses

None - Do not eat the doses indicated.

**Table 3:** Demonstration of the Log-linear class for categorized data analysis regarding depression as a response variable related to the other variables.

Variable	Depression level	*p-*value	OR	95% CI
Age	Mild	0.96	0.97	[0.32; 2.91]
	Moderate	**0.042**	0.34	[0.12; 0.96]
	Severe	0.27	0.64	[0.29; 1.40]
Viral Load	Mild	0.77	1.38	[0.16; 12.13]
	Moderate	0.73	1.46	[0.17; 12.88]
	Severe	0.68	1.42	[0.27; 7.36]
Lymphocytes T CD4+	Mild	0.07	2.56	[0.92; 7.09]
	Moderate	0.50	0.70	[0.25; 1.97]
	Severe	0.68	0.85	[0.40; 1.79]
Marital status	Mild	0.46	0.69	[0.26; 1.84]
	Moderate	0.98	1.01	[0.35; 2.91]
	Severe	0.88	1.06	[0.48; 2.33]
Education	Mild	0.91	0.94	[0.35; 2.51]
	Moderate	0.15	2.16	[0.76; 6.10]
	Severe	0.99	0.99	[0.47; 2.09]
Transmission	Mild	0.72	0.84	[0.31; 2.25]
	Moderate	0.60	0.76	[0.27; 2.13]
	Severe	**0.076**	0.51	[0.24; 1.07]
Orientation	Mild	1.00	1.00	[0.38; 2.61]
	Moderate	0.11	0.42	[0.14; 1.23]
	Severe	0.17	0.59	[0.28; 1.28]
Ethinicity	Mild	0.76	0.78	[0.16; 3.74]
	Moderate	0.22	0.42	[0.11; 1.67]
	Severe	0.25	3.32	[0.42; 26.00]
Adherence	Mild	0.53	0.60	[0.12; 2.99]
	Moderate	**0.046**	0.26	[0.07; 0.97]
	Severe	0.43	0.61	[0.18; 2.09]

**Table 4:** Chances of individual develop depression in the studied group. Observed distribution of depression per transmission and adhrence.

		Depression				
Transmission	Adherence	Minimum	Mild	Moderate	Severe	Total
Same Sex	Yes	54 (64.3%)	17 (20.2%)	7 (8.3%)	6 (7.1%)	84
	No	4 (40%)	3 (30%)	1 (10%)	2 (20%)	10
Oposite Sex/Others	Yes	82 (73.2%)	14 (12.5%)	6 (5.4%)	10 (8.9%)	112
	No	6 (60%)	1 (10%)	3 (30%)	0 (0%)	10

**Table 5:** Demonstration of the multivariate analysis regarding adherence as a response variable related to the other variables studied.

Variable	*p-*value	Odds Ratio	95% CI
Age	0.33	1.85	[0.61; 5.60]
Ethnicity	0.0008	7.68	[2.34; 25.21]
Marital status	0.47	1.6	[0.44; 5.78]
Education	0.53	0.98	[0.93; 1.04]
Transmission	0.28	2.18	[0.53; 8.94]
Sexual Orientation	0.37	0.5	[0.11; 2.26]
Lymphocytes T CD4^+^	0.5	1.45	[0.53; 8.94]
Viral load HIV	0.26	0.35	[0.06; 2.17]
Depression	0.0134	3.81	[1.32; 11.02]

For the regression model for a variable adherence, the above results for all variables tested. Among them, only ethnicity and depression are statistically significant (*p*<0.05). The results can be interpreted through odds ratios and their respective confidence intervals.

The interpretation of the odds ratios indicates that, from the statistical model, it is considered that black men have a 7.7 times greater chance of not being adherent in relation to men of the other ethnicities. Men who have any level of depression have a 3.8 times greater chance of not being adherent compared to those without depression.

**Table 6:** Observed distribution of adherence per ethnicity and depression.

Variable		Adherence		
Ethinicity	Depression	Adherence	non adherence	Total
White/Mixed	w/o depression	125 (94%)	8 (6%)	133
	w depression	58 (90.6%)	6 (9.4%)	64
Black	w/o depression	11 (84.6%)	2 (15.4%)	13
	w depression	2 (33.3%)	4 (66.7%)	6
